# From Plants to Birds: Higher Avian Predation Rates in Trees Responding to Insect Herbivory

**DOI:** 10.1371/journal.pone.0002832

**Published:** 2008-07-30

**Authors:** Elina Mäntylä, Giorgio A. Alessio, James D. Blande, Juha Heijari, Jarmo K. Holopainen, Toni Laaksonen, Panu Piirtola, Tero Klemola

**Affiliations:** 1 Section of Ecology, Department of Biology, University of Turku, Turku, Finland; 2 Department of Environmental Science, University of Kuopio, Kuopio, Finland; University of Bristol, United Kingdom

## Abstract

**Background:**

An understanding of the evolution of potential signals from plants to the predators of their herbivores may provide exciting examples of co-evolution among multiple trophic levels. Understanding the mechanism behind the attraction of predators to plants is crucial to conclusions about co-evolution. For example, insectivorous birds are attracted to herbivore-damaged trees without seeing the herbivores or the defoliated parts, but it is not known whether birds use cues from herbivore-damaged plants with a specific adaptation of plants for this purpose.

**Methodology:**

We examined whether signals from damaged trees attract avian predators in the wild and whether birds could use volatile organic compound (VOC) emissions or net photosynthesis of leaves as cues to detect herbivore-rich trees. We conducted a field experiment with mountain birches (*Betula pubescens* ssp. *czerepanovii*), their main herbivore (*Epirrita autumnata*) and insectivorous birds. Half of the trees had herbivore larvae defoliating trees hidden inside branch bags and half had empty bags as controls. We measured predation rate of birds towards artificial larvae on tree branches, and VOC emissions and net photosynthesis of leaves.

**Principal Findings and Significance:**

The predation rate was higher in the herbivore trees than in the control trees. This confirms that birds use cues from trees to locate insect-rich trees in the wild. The herbivore trees had decreased photosynthesis and elevated emissions of many VOCs, which suggests that birds could use either one, or both, as cues. There was, however, large variation in how the VOC emission correlated with predation rate. Emissions of (*E*)-DMNT [(*E*)-4,8-dimethyl-1,3,7-nonatriene], β-ocimene and linalool were positively correlated with predation rate, while those of highly inducible green leaf volatiles were not. These three VOCs are also involved in the attraction of insect parasitoids and predatory mites to herbivore-damaged plants, which suggests that plants may not have specific adaptations to signal only to birds.

## Introduction

When attacked by invertebrate herbivores plants emit an assemblage of chemical signals (i.e. infochemicals), which attract predators and parasitoids of the herbivores [1]–[6]. Novel herbivore-induced volatile organic compounds (VOCs) are particularly significant in the attraction of invertebrates such as predatory mites, entomopathogenic nematodes and hymenopteran parasitoids e.g. [1], [ 2], [ 5]–[11]. However, much less is known about mutualistic interactions, in particular about potential signalling, between plants and vertebrate predators. Cues received by insectivorous birds are interesting because birds may compete with invertebrate predators and parasitoids for the same prey or hosts (e.g. caterpillars). It has even been suggested that predators would be more profitable to plants than parasitoids because they remove the herbivore immediately from the plant [12], [13].

Birds can use visible feeding marks in leaves or qualitative structural differences among plant individuals as cues to find insect herbivores [14]–[17]. Behavioural experiments conducted in the aviary have shown that insectivorous birds may also locate their prey using signalling cues from plants, even if they cannot see the herbivores or the defoliated plant parts [18], [19]. However, the idea that birds or any other vertebrates can use indirect information as an indicator of herbivore presence on the plant has not been tested in nature.

Since avian predation can reduce herbivore load or damage of plants considerably e.g. [20]–[23], we hypothesize an adaptive advantage for plants that attract avian predators that reduce their herbivore load (i.e. a “cry-for-help”). However, predators or parasitoids may have adapted to use cues from herbivore damaged plants without plants specifically adapting for this purpose [24]–[26]. For example, such cues can be products or by-products of induced chemical defence, or other structural, physiological or chemical changes in the plant that are sensed by natural enemies of the herbivores. It is therefore crucial to understand the mechanisms behind the attraction before reaching conclusions about potential co-evolution between plants and avian predators of their herbivores [11], [27].

The two primary sensory mechanisms that birds could be using to detect plants carrying herbivores are vision and olfaction. Light reflection from intact leaves of silver birch (*Betula pendula*) is different between defoliated and intact trees throughout the visual range of passerine birds, suggesting that birds might be able to detect herbivore-damaged trees visually [19]. Diurnal birds have a wide range of vision (315–700 nm), and they can probably distinguish more shades of colour than humans [28], [29]. Birds have four cone cell types and colour-vision-enhancing oil droplets in their eyes, and thus a tetrachromatic vision, compared to the trichromatic vision of humans [28], [29]. The olfactory ability of most birds, including passerines, was long thought to be negligible [30]. However, recent studies have shown that passerines can use their olfaction in many situations, including during foraging, in aromatising nests and recognizing predators [30]–[34]. It is therefore possible that olfaction could also be utilized by birds in receiving signals from plants.

In two previous aviary experiments using undamaged detached branches we showed that birds were attracted either by olfactory or visual reflectance cues from the host plants, or a combination of these. The birds were not presented with any herbivores, or their faeces or leaf remnants that could have provided strong odorous cues [18], [19]. In this study we proceeded on from the aviary experiments by conducting a field experiment to investigate whether birds in the wild are attracted to plants that carry herbivores, even if the herbivores and damaged plant parts are hidden from view. Specifically, we measured whether the daily predation rates on artificial larvae were higher in trees with ongoing insect defoliation, but with real larvae unreachable inside bagged branches, than in control trees with only empty bags on branches. Since the birds could not see the real larvae or the damaged leaves during defoliation, we again inferred that a potential interest of birds in herbivore-damaged trees must arise from either olfactory or visual reflectance cues from the host trees.

In addition, we measured amounts of VOCs emitted by damaged and undamaged trees to determine differences in the volatile profile that may be detected by foraging birds. Novel VOCs emitted from herbivore-damaged birch leaves may be the first indicators of herbivore presence to predators. We also measured net photosynthesis of the same experimental trees to reveal potential grounds for visual cues after leaf damage [35]–[37]. The visual cues may be related to photochemical reflectance, which has been observed to change along with CO_2_ assimilation; it is thus closely related to the VOC emissions and could explain the changes in the plant-herbivore-predator system.

## Methods

### Study site and design

**Figure 1:**
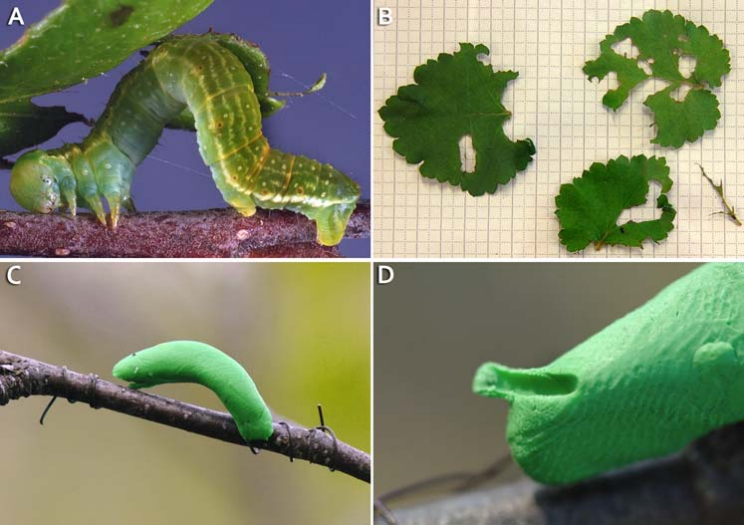
Photos of the real and the artificial larvae. A) A fifth instar *Epirrita autumnata* larva on a branch. B) Larval feeding damage on mountain birch (*Betula pubescens* ssp. *czerepanovii*) leaves. C) A plasticine larva on a mountain birch branch. D). A beak marking on a plasticine larva indicating a predation attempt by an insectivorous bird.

The experiment was conducted at the Kevo Subarctic Research Station (69°45′N, 27°01′E) in northernmost Finland, in June 2007. We selected 30 mountain birch trees (*Betula pubescens* ssp. *czerepanovii* (Orlova) Hämet-Ahti) from the mountain birch forest close to the station. These trees did not have any obvious herbivore damage prior to the experiment. Mountain birches at Kevo are typically poly-cormic, i.e. they are bush-like formations with multiple stems called ramets [38]. All the ramets of a bush belong to the same individual and genotype. From each experimental tree we selected one ramet. We chose the trees so that they could be arranged in pairs that had selected ramets of similar phenotype (height 2–4 m and similar trunk diameter) and so that the trees grew close to each other (2–10 m). The pairing was done to get two similar groups of trees and was not used in further analyses. Within tree pairs, we randomly selected trees which were to be herbivore trees with autumnal moth larvae [*Epirrita autumnata* (Borkhausen), Lepidoptera, Geometridae; Fig. 1A, B], and which were to be control trees without larvae. Autumnal moths are the main herbivores of these trees in the study region, occasionally causing large scale forest defoliation [39]. We placed mesh bags (c. 80×35 cm, mesh 0.4 mm) on three branches of the selected ramet of each experimental tree. In the herbivore trees we enclosed 20 laboratory-hatched third-instar autumnal moth larvae (corresponding to a high natural abundance [40]) inside each bag, while bags on control trees were left empty. We placed the bags and larvae on the first 14 trees (seven pairs) on 8^th^ June and to the last 16 trees (eight pairs) on 9^th^ June. These dates mark the start of defoliation. The larvae were allowed to feed on the leaves inside the bags throughout the experiment. At the end of the experiment, the larvae were in their last (5^th^) instar.

On 10^th^ June we placed ten artificial larvae (Fig. 1C) on the selected ramet of each tree. The artificial larvae were made of light green plasticine (close to the natural colour of real autumnal moth larvae) and thin, reddish-brown metal wire (Ø 0.35 mm). The plasticine larvae were approximately the size of a big fifth instar autumnal moth larva (length 2–3 cm, Ø 3–4 mm). The plasticine larvae were placed randomly on branches of the selected ramets close to bagged branches of all experimental trees and fastened in position with the metal wire. From the next day onwards we checked the condition of these plasticine larvae daily and replaced damaged with new ones attached to a slightly different place (E.M. did this in each case). A plasticine larva was determined as damaged by birds if it had some marks that were consistent with bird pecking damage (Fig. 1D) and could not be explained otherwise (e.g. not a scratch by a nearby branch). After seven days (17^th^ June) we replaced all the plasticine larvae with new ones. We removed all plasticine larvae from the trees on 24^th^ June when the number of plasticine larvae damaged by birds per day had diminished. In the study year there were practically no wild autumnal moth larvae in the area as it was a bottom year for this cyclic herbivore [41]. As we did not discover herbivore damage by other insect herbivores in any of the trees, it was safe to assume that the control trees did not show a significant response to leaf herbivory during the experiment.

We visually estimated the loss of foliage inside the mesh bags of herbivore trees one week from the start of defoliation (15^th^ June) and at the end of the experiment (25^th^ June). On 15^th^ June the defoliation was 5–20% (mean±SD was 11.0±4.7%) and on 25^th^ June 10–60% (mean±SD was 34.0±14.8%).

The study area was ca. 50×50 m in size. In that area and in its surroundings we observed local birds (mainly singing males) on 12^th^ June. There were territories of at least four pied flycatchers (*Ficedula hypoleuca*), three willow warblers (*Phylloscopus trochilus*), three bramblings (*Fringilla montifringilla*), one great tit (*Parus major*) and one Siberian tit (*Parus cinctus*). None of the birds observed had its nest in the middle of the study area. During other days we also observed common redpolls (*Carduelis flammea*), yellow wagtails (*Motacilla flava*), bohemian waxwings (*Bombycilla garrulus*), bluethroats (*Luscinia svecica*) and fieldfares (*Turdus pilaris*) in the study area. At the time of our experiment most of the local birds were in the early stages of nesting (i.e. egg laying, incubating or feeding young nestlings) and thus in need of ample food resources.

### Volatile organic compounds

We collected VOCs [homo-, mono-, sesquiterpenes and green leaf volatiles (GLV)] from 14 tree pairs six days (14^th^–15^th^ June) after the start of defoliation, and from seven tree pairs 10–11 days (18^th^ June) after the start of defoliation. For each tree pair one branch from each tree was sampled concurrently. One mesh bag from each tree was opened immediately before sampling. The feeding larvae were removed from the outermost part of the defoliated branch to be sampled and moved further back. Both mesh bags (defoliated and control) were refastened once the branch area to be sampled was exposed, so that feeding larvae could not escape. Polyethylene terephthalate (PET) bags (size 45×55 cm, LOOK, Terinex Ltd, Bedford, England) were pre-heated for 1 h at+120°C before collections, to prevent any contamination from the bag, and subsequently cooled. These bags were carefully added to branches and fastened securely to the bark of a branch taking care not to damage any foliage. One of the two outermost bag corners was cut, and an air inlet tube and a sensory unit of a HOBO Micro Station Data Logger (MicroDAQ.com Ltd, Contoocook, NH, USA) for recording climatic data were inserted and supported by a tripod. Clean charcoal-filtered and MnO_2_ scrubbed air was pumped at the rate of 600 ml min^−1^ through Teflon tubing to flush the system, and then reduced to 230 ml min^−1^. The remaining bag corner was cut and a stainless steel tube containing approximately 150 mg of Tenax TA-adsorbent (Supelco, mesh 60/80) was inserted and fastened into position. Air was pulled through the Tenax tube by battery-operated sampling pumps (Rietschle Thomas, Puchheim, Germany). The air flow through the Tenax tube was set to 200 ml min^−1^ with an M-5 bubble flowmeter (A.P. Buck, Orlando, FL, USA). The VOC collection system including inlet and outlet pumps, clean air filters, HOBO Micro Station Data Logger and batteries was installed into a portable plastic toolbox. During the sampling period (60 minutes for the first collection series, and 30 minutes for the second series), the temperature, photosynthetically active radiation (PAR) and air humidity inside the plastic bags were monitored with a HOBO Micro Station Data Logger.

The VOC samples were analysed with a gas chromatograph-mass spectrometer (Hewlett-Packard GC 6890, MSD 5973). Trapped compounds were desorbed with a thermal desorption unit (Perkin-Elmer ATD400 Automatic Thermal Desorption system) at 250°C for 10 min, cryofocused at −30°C, and injected onto a HP-5 capillary column (50 m×0.2 mm i.d.×0.5 µm film thickness, Hewlett-Packard) with helium as a carrier gas. The oven temperature program was held at 40°C for 1 min and then raised to 210°C at a rate of 5°C min^−1^, and finally further to 250°C at a rate of 20°C min^−1^. The compounds (mono-, homo-, and sesquiterpenes and green leaf volatiles, GLVs) were identified by comparing their mass spectra with Wiley library and pure standards. Emissions were presented in ng cm^−2^ h^−1^. As biogenic emissions depend strongly on light and temperature all terpene emissions (not GLVs) were made comparable by standardizing them to a temperature of 30°C using the classic algorithm established by [42]. We used the temperature coefficient (β[K^−1^]) of 0.09 recommended by [42] to standardize monoterpene emissions, and the β[K^−1^] of 0.18 as used by [43] to standardize sesquiterpene emissions.

### Photosynthesis

We measured the leaf photosynthesis of the herbivore and control trees with a CI-510 Portable Photosynthesis System (CID, Inc, Vancouver, WA, USA) after VOC sampling on the 14^th^ day since the start of larval feeding (22^nd^ or 23^rd^ June, depending on the start date). We used nine herbivore trees and eight control trees. The measured leaves were outside the mesh bags on adjacent branches as they were the leaves that the birds could see. We used 2–3 leaves per tree. Each chosen leaf was fully expanded and well exposed to light. It was clamped in a gas-exchange cuvette and subjected to the natural sun light and temperature conditions at the time of sampling. An Infra Red Gas Analyser (IRGA), close to the leaf cuvette, detected the differential concentration of CO_2_ between the inside of the cuvette and the incoming reference air. During each measurement the reference air was positioned away from the user to avoid any unexpected variation.

### Statistical analyses

We modelled the probability of a predation event using the events/trials syntax for binomial data, i.e. damaged plasticine larvae from preset larvae was the response variable in statistical tests of the bird predation experiment. We conducted the tests with generalized linear models (the GENMOD procedure of the SAS statistical software, version 9.1) using logit link functions. Specifically, we tested whether the probability of a predation event was affected by any of the following fixed factors throughout the whole duration of the experiment: treatment (herbivore or control), time (days since the start of defoliation) or time^2^ (in order to test the quadratic effect of time). We also included the interaction terms of *treatment*×*time*, *treatment*×*time^2^*. With separate analyses, we tested whether differences in the defoliation percentage could explain the probability of a predation event in the selected ramets of the herbivore trees alone. The influences of defoliation percentage after one week and at the end of the experiment were tested separately matching to two separate VOC samplings. In order to assume complete independence across the subjects, the tree was used as a subject and a repeated effect in the REPEATED statement in all analyses.

**Figure 2:**
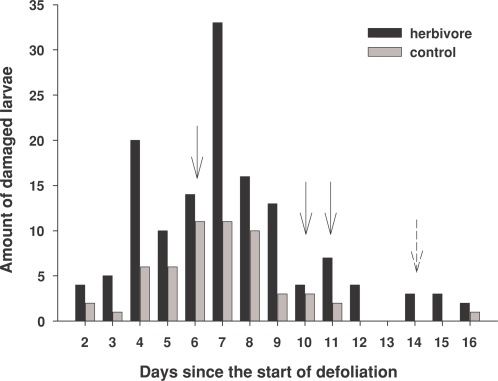
The daily numbers of damaged plasticine larvae found from herbivore (black bars) and control (grey bars) birches. The X-axis shows the number of days since the start of defoliation by autumnal moth larvae in the herbivore trees. Solid and hatched arrows show the days when the volatile organic compounds (VOCs) and net photosynthesis rate, respectively, were measured.

A general linear model (the GLM procedure of the SAS) was used to analyse differences in emissions of each VOC (in total 15 compounds) between control and herbivore birches. Explanatory factors were treatment, sampling date and their interaction for the first collection series and treatment alone for the second series. However, sampling date (*p*>0.087 for all VOCs analysed) or the interaction (*p*>0.067 for all VOCs analysed) were not significant. Thus, we report the results only for the treatment effect. Further, Spearman's rank correlation (the CORR procedure of the SAS) between the total sum of damaged plasticine larvae per tree and the emission of each VOC (in total 28 trees) was used to analyse which compounds may provide the most important cues for birds. As VOCs were often correlated among each other, which would have obscured statistical and inferential interpretation [44], we used a simple correlative approach instead of e.g. a generalized linear model with all 15 VOCs concurrently as explanatory variables. A transformation of number of correlated variables into a smaller number of uncorrelated principal components was not desired either, because we wanted to identify single compounds with potential to explain bird attraction. An appropriate use of principal components would also have required a substantially larger sample size than was possible to collect in this study [45]. The VOC data from the first sampling were used for the correlation analysis because the time of sampling closely matched the peak of bird predation (Fig. 2).

A general linear mixed model (the procedure MIXED of the SAS) was used to analyse net photosynthesis at the end of the experiment. The fixed explanatory factors were treatment, day of measurement (22^nd^ or 23^rd^ June) and their interaction. As before, the tree was used as a subject and a repeated effect in the REPEATED statement.

## Results

There were clearly more damaged plasticine larvae on the herbivore trees than on the control trees (Table 1, Fig. 2). The probability of the predation event (back-transformed least square mean estimate from the logit scale) was 0.045 (*95% CI* = 0.031 to 0.064) in the herbivore trees and 0.018 (*95% CI* = 0.012 to 0.027) in the control trees. The odds ratio for treatment (herbivore vs. control) was significant (*OR* = 2.59, *95% CI* = 1.61 to 4.16). The amount of damaged plasticine larvae increased at first, reaching a peak on the sixth day from the introduction of the plasticine larvae (i.e. the seventh day from the start of herbivory) (Table 1, Fig. 2). After that the interest decreased gradually (Fig. 2). There was no significant interaction between the treatment and time or treatment and time^2^ (Table 1). Neither the defoliation percentage after one week (*χ^2^* = 0.63, *df* = 1, *p* = 0.43) nor at the end of the experiment (*χ^2^* = 1.87, *df* = 1, *p* = 0.17) affected the probability of predation of plasticine larvae on the herbivore trees, indicating that the increased defoliation did not increase the attraction of birds to trees.

**Table 1:** Results of the generalized linear models on factors affecting the probability of predation event of plasticine larvae.

Final model	DF	χ^2^	*p*
*treatment*	1	7.21	0.0072
*time*	1	11.38	0.0007
*time^2^*	1	13.79	0.0002

Removed effects	DF	χ^2^	*p*
*time×treatment*	1	0.56	0.456
*time^2^×treatment*	1	0.93	0.335

The analysis was first based on a full model, from which effects were dropped one by one in order of least significance. The final model is given with the statistically significant (*p*<0.05) variables. Results for the other factors are given when they were added alone to the final model.

**Figure 3:**
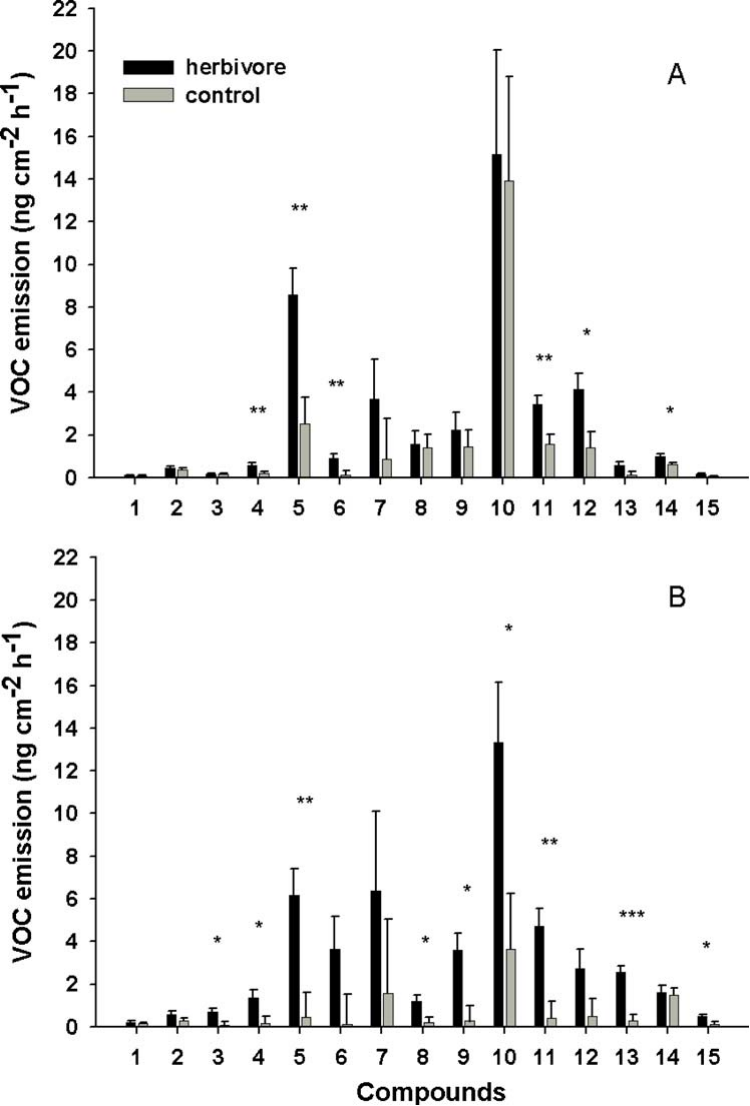
The volatile organic compound (VOC) emissions from herbivore (black bars) and control (grey bars) birch branches (ls means+SE from statistical models are shown). A) six days, *n* = 14 in both control and herbivore trees, and B) 10–11 days since the start of defoliation by autumnal moth larvae, control: *n* = 7 and herbivore: *n* = 6. Compounds: (1) α-pinene, (2) β-myrcene, (3) limonene, (4) β-ocimene, (5) linalool, (6) (*E*)-DMNT, (7) α-copaene, (8) α-humulene, (9) caryophyllene oxide, (10) (*E*)-β-caryophyllene, (11) β-bourbonene, (12) cis-3-hexenyl acetate, (13) cis-3-hexen-1-ol+(*E*)-2-hexenal, (14) nonanal, (15) cis-3-hexenyl butyrate. (*: *p*<0.05; **: *p*<0.01; ***: *p*<0.001).

Defoliation of mountain birch by autumnal moth larvae led to significant induction of several VOCs from the defoliated ramet. Six days after the onset of defoliation, feeding damage significantly induced the emission of β-ocimene (compound #4 in Fig. 3), linalool (#5), (*E*)-DMNT [(*E*)-4,8-dimethyl-1,3,7-nonatriene] (#6), β-bourbonene (#11), cis-3-hexenyl acetate (#12) and nonanal (#14), as compared to undamaged controls (Figure 3A). In the second sampling (10–11 days of defoliation), significant induction of limonene (#3), β-ocimene (#4), linalool (#5), α-humulene (#8), caryophyllene oxide (#9), (*E*)-β-caryophyllene (#10), β-bourbonene (#11), cis-3-hexen-1-ol+(*E*)-2-hexenal (#13) and cis-3-hexenyl butyrate (#15) emissions was also observed (Fig. 3B). A few seemingly clear inductions were not statistically significant due to high within-treatment variance and relatively low sample size, especially in the second sampling. Such compounds were α-copaene (#7), cis-3-hexen-1-ol+(*E*)-2-hexenal (#13) and cis-3-hexenyl butyrate (#15) in the first sampling, and (*E*)-DMNT (#6), α-copaene (#7) and cis-3-hexenyl acetate (#12) in the second sampling.

**Figure 4:**
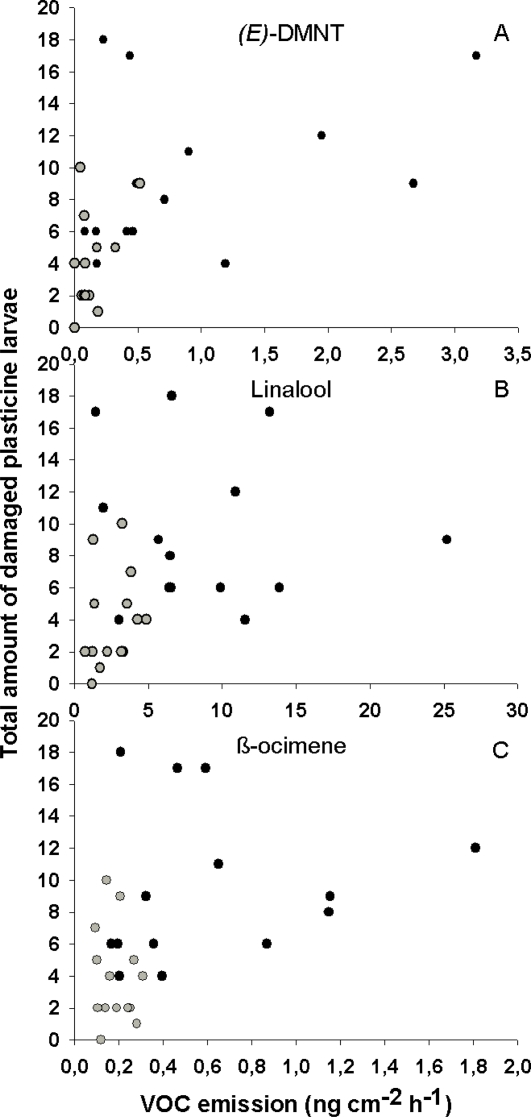
Scatter plots of three volatile organic compounds (VOCs) and the total sum of damaged plasticine larvae in herbivore (black dots) and control (grey dots) trees (*n* = 28). A) (*E*)-DMNT, B) linalool and C) β-ocimene. Note the different x-axes in the panels.

The sum of damaged larvae per tree was strongly positively correlated with the amounts of (*E*)-DMNT (#6), β-ocimene (#4) and linalool (#5) emitted from the herbivore damaged leaves (Table 2, Fig. 4), which suggests that these three compounds are prominent candidates for being volatile cues that attract birds to the trees. The other measured VOCs, including those induced by herbivory, had no obvious correlations with the number of damaged larvae (Table 2).

**Table 2:** Spearman's rank correlation coefficients (*r*_S_) between individual volatile organic compound emissions in the first measurement (6 days after the start of defoliation) and the total sum of damaged plasticine larvae per tree (*n* = 28 trees) in both herbivore and control trees.

Compound	No.	Group	r_S_
*(E)-DMNT*	#6	homoterpene	0.576**
*β-ocimene*	#4	monoterpene	0.454*
*linalool*	#5	monoterpene	0.454*
*β-bourbonene*	#11	sesquiterpene	0.242
*cis-3-hexen-1-ol+(E)-2-hexenal*	#13	green leaf volatile	0.224
*cis-3-hexenyl butyrate*	#15	green leaf volatile	0.162
*α-pinene*	#1	monoterpene	0.160
*α-copaene*	#7	sesquiterpene	0.147
*cis-3-hexenyl acetate*	#12	green leaf volatile	0.142
*(E)-β-caryophyllene*	#10	sesquiterpene	0.093
*nonanal*	#14	green leaf volatile	0.080
*limonene*	#3	monoterpene	−0.012
*caryophyllene oxide*	#9	sesquiterpene	−0.015
*α-humulene*	#8	sesquiterpene	−0.023
*β-myrcene*	#2	monoterpene	−0.107

Column ‘No.’ refers to the number of the compound in Figure 2. Column ‘Group’ indicates into which group of VOCs the compound belongs. (^*^: *p*<0.05; ^**^: *p*<0.01).

At the end of the experiment (14 days from the start of defoliation), there was significantly more leaf photosynthesis (µmol m^−2^ s^−1^) outside the mesh bags on control trees than on herbivore trees (*F_1,13_* = 14.07, *p* = 0.0024; *herbivore*: *ls mean* = 2.38, *95% CI* = 1.09 to 3.68; *control*: *ls mean* = 5.64, *95% CI* = 4.28 to 7.00). The measurement day (*F_1,13_* = 3.09, *p* = 0.10) or its interaction with treatment (*F_1,13_* = 0.08, *p* = 0.78) did not have an effect on the amount of net photosynthesis.

## Discussion

Our results provide the first support that passerine birds in a natural setting use cues other than visual recognition of herbivore larvae, damaged leaves or larval faeces to locate insect-rich trees. The number of damaged plasticine larvae was notably greater on the herbivore trees than on the control trees, which indicates that passerine birds were foraging more on mountain birches that had hidden defoliation by autumnal moth larvae than on control trees with no herbivory. For the first few days of the experiment, birds displayed moderate interest in the plasticine larvae. This was followed by a clear peak after which interest diminished gradually (Fig. 2). It was expected that the local birds would lose interest in the plasticine larvae when they learned that they were inedible.

There were rather strong positive correlations between certain VOCs and the amount of damaged larvae found on the trees. The three VOCs that had the highest correlation coefficients [(*E*)-DMNT (#6), β-ocimene (#4) and linalool (#5)], were emitted significantly more from herbivore than control trees in the first measurement, which was made at the time of the highest avian predation rate (6 days of defoliation; Fig. 2). These compounds belong to a group of VOCs that are also highly significantly induced by herbivory in other herbivore-damaged woody plants [46], [47]. Thus, it can be assumed that the passerine birds could use these VOCs to find insect-rich trees. They are also among key compounds in the attraction of insect parasitoids and predatory mites to herbivore-damaged plants [7], [8], [48], [49] and suggested to be the most promising candidates for aerial cues in plant-mediated communication [50]. This suggests that birds may be using the same cues from the trees as invertebrate predators and parasitoids, and that plants may not have specific adaptations for signalling just to birds. This is an important finding for understanding co-evolution in the multi-trophic interactions between plants, herbivores and the natural enemies of herbivores. However, it must be noted that a correlation does not confirm causality. It is therefore possible that other correlated factors induced by herbivory could explain the higher predation rates. Our results nevertheless indicate that (*E*)-DMNT (#6), β-ocimene (#4) and linalool (#5) are the strongest nominees for the causal cue that birds use, and therefore the compounds that should be considered in future experimental studies. This is supported by the fact that the amount of green leaf volatiles (GLVs) was not correlated with plasticine larvae predation rates, although their emission was also significantly induced by larval feeding. In this experiment birds may also have smelled the larval faeces inside the bags but that does not explain the correlation between VOC emissions and the predation rate. It would not explain the previous findings from behavioural experiments either [18], [19].

The VOCs emitted in the greatest quantities were (*E*)-β-caryophyllene (#10), linalool (#5) and cis-3-hexenyl acetate (#12), which has also been observed to be induced by herbivore damage in silver birch [47]. The similar amounts of (*E*)-β-caryophyllene (#10) (average of 39% of the total emission) in the control and herbivore-damaged birch leaves in the mid-experiment but significantly higher amounts in the damaged leaves at the end of the experiment might reflect the effect of long-term biochemical changes in the leaves. Dominant constitutively emitted compounds, e.g., (*E*)-β-caryophyllene (#10), did not seem to be important in the attraction of birds or other herbivores to the plants, even though (*E*)-β-caryophyllene is induced by herbivore feeding in several plant species and is a major attractant e.g. of entomopathogenic nematodes [9] and predatory mites [51]. The VOCs that had the highest correlations with avian predation rates, such as (*E*)-DMNT (#6) (discussed above), had relatively low emission rates, particularly from intact control plants. The possible genetic differences between the tree individuals [all mountain birches are some level hybrids of downy birch (*B. pubescens*) and dwarf birch (*B. nana*)] can affect the amount of VOCs released after a certain level of defoliation. This threshold-based operation is supported because increased defoliation percentage inside the mesh bags did not alone increase the predation rate of plasticine larvae.

Our results indicate that the methods used by birds to find insect-rich trees could be either olfactory through volatile organic compounds or visual through changes in photosynthetic reflectance, or both. Our earlier aviary experiments exclude the possibility that birds only use cues directly from herbivores and also show that at least the three tested bird species [willow warblers, great tits and blue tits (*Cyanistes caeruleus*)] behaved similarly [18], [19]. The measurements of VOC emissions show clear differences between herbivore and control trees, which coupled with our observations of damaged plasticine larvae, suggest that olfaction is a noteworthy candidate mechanism for birds to locate herbivore-damaged trees. Nevertheless, there was also a significant reduction in the amount of net photosynthesis in herbivore-damaged trees at the end of the monitoring period. Reduced photosynthesis could cause differences in the colour and reflectance of the leaves [35]–[37]. In accordance with this, our previous results with silver birch showed that the reflectance of leaves after defoliation by autumnal moth larvae was higher in control trees than in herbivore trees even when the defoliation had already ceased and the larvae had pupated [19]. There the control trees had higher reflectance in the entire visible spectrum of passerine birds, not only in UV wavelengths. In fact, the difference was greatest in the wavelengths of green light (ca. 500 nm). That matches well with the differences in the net photosynthesis observed at the end of the monitoring period in this study. Yet another possibility is that the birds can somehow see the VOCs, many of which readily form secondary aerosols in the atmosphere [52], finally leading to a blue haze formation. The low concentrations of in-situ produced VOCs, especially in cold climatic conditions, can however make it difficult for insectivorous birds to use them alone as cues, especially upwind of the insect-rich trees. Thus, it can be speculated if they use both vision and olfaction, depending on the environmental conditions. Nevertheless, this experiment confirmed that birds distinguish between herbivore and control trees in the wild, and provides preliminary support for volatile organic compounds as a potential candidate mechanism.
